# The effect of expectancy on conditioned pain modulation: evidence from functional near-infrared spectroscopy

**DOI:** 10.3389/fpsyg.2025.1525216

**Published:** 2025-03-17

**Authors:** Xueshan Li, Min Liu, Bo Liu, Heng Yue, Xiangjuan Cheng, Hugejiletu Bao

**Affiliations:** ^1^School of Psychology, Inner Mongolia Normal University, Hohhot, China; ^2^School of Journalism and Communication, Xiamen University, Xiamen, China; ^3^The Psychological Health Education Centre, Anhui Polytechnic University, Wuhu, China; ^4^College of Physical Education, Inner Mongolia Normal University, Hohhot, China

**Keywords:** pain, conditioned pain modulation, expectancy, attention, prefrontal cortex, functional near-infrared spectroscopy

## Abstract

**Background and objective:**

The psychological mechanisms that make Conditioned Pain Modulation (CPM) an effective non-pharmacological intervention are still not fully understood. Expectancy is believed to be a critical psychological factor affecting CPM effects, but its specific role has yet to be fully clarified. This study aims to explore the relationship between expectancy and CPM while providing physiological evidence using functional near-infrared spectroscopy (fNIRS).

**Method:**

A standardized CPM induction paradigm was implemented, with verbal guidance used to induce expectancy. The Numeric Rating Scale (NRS) assessed the intensity of the test stimulus (TS), while an 11-point scale evaluated participants’ attentional focus on the TS and the effect of expectancy. fNIRS was employed to monitor changes in prefrontal cortex (PFC) activity.

**Results:**

Expectancy significantly amplified the CPM effect (*p* = 0.036) while markedly reducing attention to the experimental stimulus (*p* = 0.004). fNIRS findings indicated significant reductions in activity within the left frontal eye field, left dorsolateral prefrontal cortex, and left frontal pole regions. In the post-test, the control group demonstrated significantly higher cortical activity in the right frontal pole region compared to the expectancy group (*p* < 0.05). Within the expectancy group, bilateral frontal pole cortical activity was significantly lower in the post-test compared to the pre-test (*p* < 0.05).

**Conclusion:**

Expectancy represents a key psychological mechanism underlying the CPM effect, potentially modulating its magnitude through attention regulation and accompanied by a reduction in oxygenated hemoglobin activity in the frontal pole region and introduced the Expectancy-Attention-CPM Modulation Model (ECAM).

## 1 Introduction

Pain is characterized as an unpleasant sensory and emotional experience tied to actual or potential tissue damage or analogous experiences (Song et al., 2020). As a multifactorial phenomenon shaped by physiological, psychological, and social influences, pain functions, from an evolutionary standpoint, as a crucial warning mechanism safeguarding the body from harm. However, maladaptive responses to pain can yield severe repercussions, manifesting as a wide range of emotional and cognitive disturbances (Moriarty et al., 2011). In addition to eliciting significant anxiety, depression, and sleep disturbances in affected individuals, pain imposes a profound economic burden on both families and society (Zhang et al., 2020).

Currently, pain management strategies are broadly categorized into pharmacological and non-pharmacological approaches. Pharmacological treatments pose significant challenges; opioids, for example, can effectively mitigate pain perception but are associated with a risk of dependency (Rieder, 2019) and may induce structural and functional changes in the brains of chronic pain sufferers (Borsook et al., 2018). Consequently, numerous non-pharmacological interventions have risen in prominence and are now widely employed in clinical settings.

Conditioned pain modulation (CPM) is identified as a mechanism underlying endogenous pain inhibition. Specifically, it describes how a pain stimulus delivered to one region of the body (conditioning stimulus, CS) can suppress the perception of pain arising from a separate stimulus applied to another region (test stimulus, TS), thereby achieving what is often referred to as an “analgesia by pain” effect. The degree of TS suppression by CS directly reflects the efficiency of an individual’s CPM mechanism; in essence, the stronger the suppression of TS by CS, the more robust the endogenous pain inhibitory capability (Tang et al., 2016).

CPM’s “top-down” approach to pain inhibition exemplifies the activation of endogenous pain regulatory mechanisms (Pud et al., 2009). The prevailing view holds that the mechanism underlying CPM involves the activation of the spinal-medullary-spinal neural circuit (Zhang et al., 2020). However, some researchers argue that the CPM effect cannot be solely attributed to the activation of this neural circuit by exogenous pain signals; rather, psychological functions also play a crucial role (Nir et al., 2012; Plinsinga et al., 2023). Among these psychological factors, expectancy is considered a potential mechanism contributing to the analgesic effect of CPM. Expectancy is defined as the anticipation of a potential outcome or the occurrence of a desired effect based on prior experiences (Deng et al., 2015). It is widely recognized as a fundamental psychological mechanism driving the placebo effect (Wei et al., 2018) and is often elicited and modulated through verbal suggestions (Cormier et al., 2013). Research indicates that positive expectancy can significantly alleviate pain, while negative expectancy (such as anticipating intensified pain) can heighten pain perception (Taylor et al., 2017). In studies exploring expectancy’s role in CPM, participants with analgesic expectancy exhibited a more pronounced CPM effect compared to those expecting heightened pain sensitivity (Cormier et al., 2013; France et al., 2016). Compared to healthy individuals, chronic pain patients generally demonstrate reduced CPM efficiency (Pickering et al., 2014). Chronic pain persistence in these patients is frequently linked to inadequate self-management, which elevates the risk of pain-related disability (Caes et al., 2021). Effective self-management strategies, however, can markedly reduce chronic pain’s impact and enhance psychological health (Nicholas et al., 2012). Positive analgesic expectancy, as an effective self-management tool, has proven to significantly mitigate pain (Shih et al., 2019).

Extensive research has delved into the neural mechanisms underpinning CPM, revealing that CPM not only reduces pain but also significantly activates the prefrontal cortex (PFC) and the periaqueductal gray (PAG; Mohr et al., 2008). Moreover, the extent of pain relief is strongly correlated with the connectivity between the dorsolateral prefrontal cortex (DLPFC) and the white matter tracts of the PAG (Bunk et al., 2020). Individual differences in PFC cortical function further influence the expression of conditioned pain modulation effects (Youssef et al., 2016), suggesting that the DLPFC may represent a pivotal brain region in the functioning of CPM.

Expectancy modulates pain through a “top-down” regulatory process, influencing both the objective processing of nociceptive signals and subjective pain perception (Ploghaus et al., 2003). Studies indicate that stimulating the dorsolateral prefrontal cortex (DLPFC) via transcranial magnetic stimulation (TMS) can enhance placebo responses, leading to a reduction in social pain (Wang et al., 2023). Notably, the right DLPFC exhibits higher levels of activation than the left (Kong et al., 2007), highlighting the DLPFC’s critical role in processing pain-related expectancy (Amanzio et al., 2013) and positioning it as a core brain region in expectancy-driven modulation.

Furthermore, the analgesic effects of endogenous opioids suggest a link between expectancy and CPM. It is hypothesized that expectancy may reduce pain perception by modulating endogenous opioid systems. Studies have demonstrated that opioid activity in regions such as the periaqueductal gray (PAG), amygdala, orbital frontal cortex (OFC), insula, left prefrontal cortex (LPFC), and anterior cingulate cortex (ACC) is closely tied to placebo effects (Wager et al., 2011), while negative expectancy or nocebo effects manifest as deactivation within these regions (Scott et al., 2008). Additionally, placebo-induced effects driven by expectancy can be blocked using the opioid antagonist naloxone (Levine et al., 1978); likewise, when patients are administered opioid antagonists, their CPM efficacy declines (King et al., 2013), and its correlation with the DLPFC weakens (Sprenger et al., 2011). However, some researchers have expressed opposing views, suggesting that the role of expectancy in CPM may not be mediated by opioids (France et al., 2016). Despite observable expectancy effects, CPM responses often remain unchanged (Nir et al., 2012), and some propose that pain relief via CPM and expectancy arises from entirely independent mechanisms (Skyt et al., 2018). In conclusion, the psychological mechanisms by which expectancy influences CPM are still debated and require deeper exploration. Variability in the methods used to induce CPM effects may account for the inconsistencies in research findings and an incomplete understanding of its underlying psychological processes.

This study therefore recruited healthy participants who were screened based on specified exclusion criteria and employed the CPM induction paradigm as recommended by the European Journal of Pain (Yarnitsky, 2010). Capsaicin-induced pain served as test stimulus, while fixed-duration cold-water immersion was used as the conditioning stimulus. Verbal suggestions were employed to induce analgesic expectancy, allowing for observation of the effects of expectancy on CPM and the activation of the PFC in both the expectancy and control groups.

## 2 Methods

### 2.1 Participants

The sample size for this study was calculated using G*Power 3.1.9.2, with parameters set at an effect size f of 0.25 and power (1–*β*) of 0.8, resulting in a target sample of 34 participants. Accordingly, Study 1 recruited 44 university students. After excluding 5 participants who did not achieve successful activation of positive expectancy, the final sample included 39 participants (18 women), divided into a control group of 19 and an expectancy group of 20. Participants’ ages ranged from 18 to 27 years (*M ± SD* = 21.95 ± 2.27).

Exclusion criteria were as follows: (1) corrected vision abnormalities; (2) ongoing pain lasting more than 3 months; (3) female participants currently in their menstrual cycle; (4) trauma or tattoos on the inner left hand or right ankle; (5) left-handedness; (6) current alcohol consumption or use of analgesics or neuroactive drugs within the past week. All participants provided informed consent and had the right to withdraw from the study at any point. Upon completion of the study, participants received psychological counseling and a compensation of 25 RMB. This study was approved by the Ethics Review Committee of Inner Mongolia Normal University.

### 2.2 Induction and assessment of conditioned pain modulation

Since the CPM effect is not contingent upon the characteristics of test stimulus, participant tolerance serves as the key criterion for evaluating the stimulus. Consequently, a range of commonly utilized pain induction methods, including thermal, mechanical, electrical, and chemical stimuli, has been incorporated (Kennedy et al., 2016). Among these methods, capsaicin-induced pain is considered a safe and non-invasive approach that reliably produces stable and lasting pain effects (Modir and Wallace, 2010). The cold pressor test (CPT) is regarded as the most potent method for inducing a conditioning stimulus (Nilsen et al., 2014) and is noted for its strong reliability (Lewis et al., 2012; Nuwailati et al., 2022). Immersion of the wrist in 12°C cold water for 1 min is a frequently recommended and widely utilized protocol (Lewis et al., 2012). Furthermore, except when applied to the same specific location on the same limb, CPM effects are generally unaffected by the stimulus site, with testing often performed on the ipsilateral or contralateral lower limbs (Damien et al., 2018).

**Figure 1:**
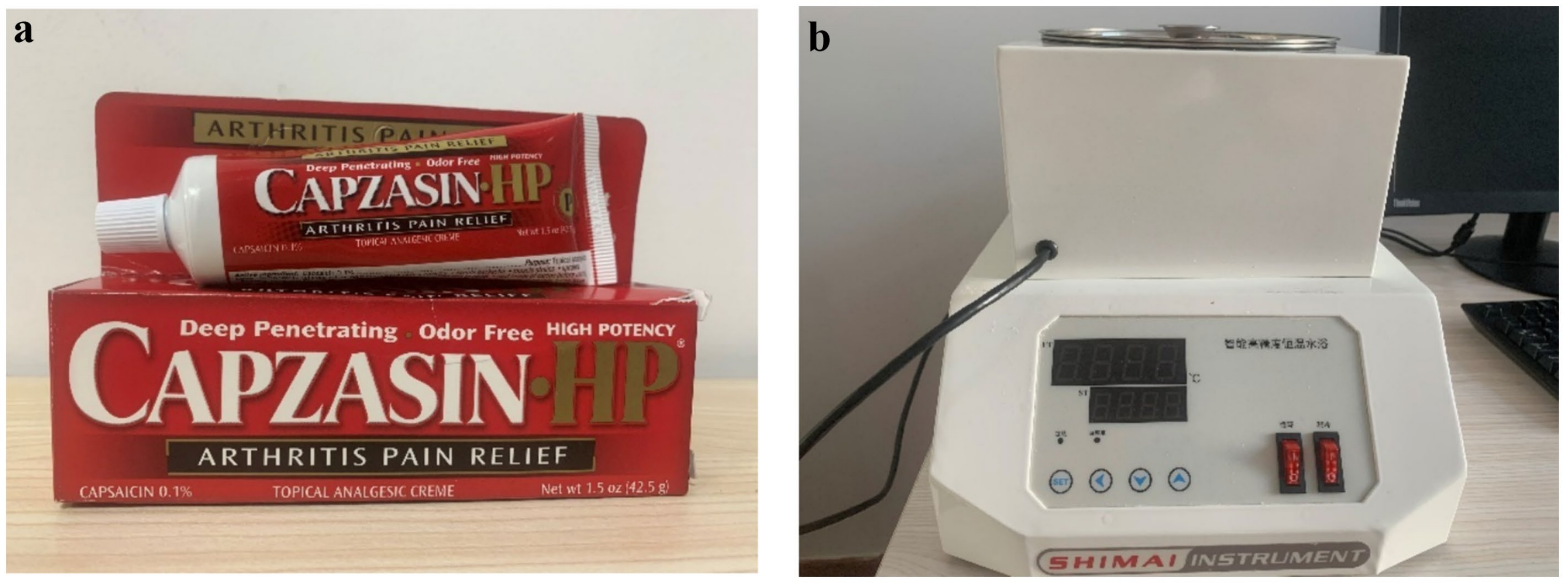
Pain induction materials, **(a)** the left image shows capsaicin ointment, **(b)** the right image shows cold water apparatus.

In this study, capsaicin application and the cold pressor test (CPT) were employed to induce test stimulus (TS) and conditioning stimulus (CS), respectively, as illustrated in Figure 1. The capsaicin utilized was a 0.1% cream, commonly used in clinical settings to relieve joint pain and sprains, as depicted in Figure 1a. The CPT was controlled using an SME–CTB high-precision low-temperature constant temperature water bath, with a temperature accuracy of ±0.01°C. The container had a diameter of 180 mm and a height of 120 mm, with a target temperature set at 12°C, maintained through continuous water circulation to ensure consistent temperature, as shown in Figure 1b. The room temperature was consistently maintained at 26°C.

The evaluation of conditioned pain effects was performed using the Numeric Rating Scale (NRS), which directly assessed the pain intensity experienced by participants on their right lower leg (experimental stimulus pain intensity). The NRS is widely recognized as an effective clinical tool for accurately and intuitively measuring pain levels (Hong et al., 2003) and has demonstrated the lowest error response rate and greatest utility in non-cross-cultural research (Atisook et al., 2021). This scale utilizes an 11-point system ranging from 0 to 10, with 0 representing no pain and 10 signifying the most severe pain imaginable or intolerable. Measurements showed that the initial pain intensity induced by capsaicin among participants was 7.58 ± 1.03, indicating successful activation of test stimulus.

### 2.3 fNIRS measurement

**Figure 2:**
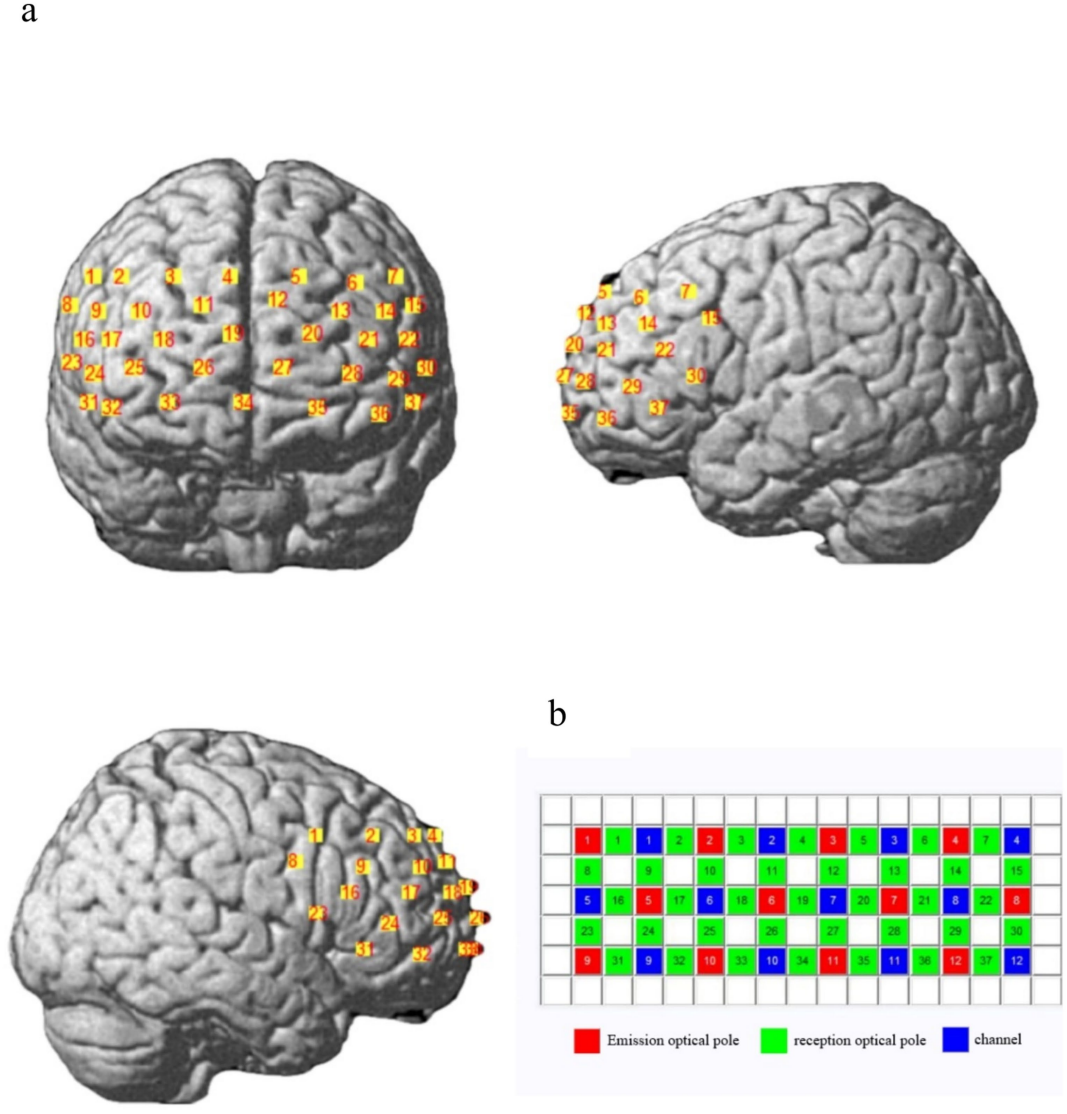
fINRS channel layout information (from left to right: anterior, left, and right views). **(a)** Position of the optical plate covering the frontal lobe. **(b)** Layout of the optical pole plate, consisting of 37 measurement channels composed of emission and reception optical poles. The red square represents the emission optical pole, the green square represents the reception optical pole, and the blue square represents the observation channel.

Functional near-infrared spectroscopy (fNIRS) is a relatively non-invasive, safe, portable, and cost-effective neuroimaging technology. By measuring light absorption, it non-invasively calculates changes in the concentrations of oxygenated hemoglobin (HbO2) and deoxygenated hemoglobin (HbR), thereby providing an indirect assessment of brain activity (Irani et al., 2007). This study utilized a LABNIRS SHIMADZU–3000 near-infrared imaging device, manufactured by Shimadzu Corporation, Japan, to record changes in participants’ cortical blood oxygen concentration. The device emits continuous waves at wavelengths of 780 nm, 805 nm, and 830 nm, with a sampling rate of 12.82 Hz. The optode array was configured in an 8 × 3 layout, comprising 12 light source emitters and 12 detectors, forming an optical array with 37 near-infrared measurement channels embedded in the optode cap (Figure 2). The spacing between adjacent emitters and detectors was 3 cm, allowing full coverage of the prefrontal cortex (Figure 2). After the experiment, a 3D digital locator by Polhemus, with a measurement accuracy of 0.01–2.94 mm, was used to capture the 3D spatial positions of the optodes on participants. Reference points were marked in the order of NZ, CZ, AL, and AR, followed by sequential collection of each emitter and detector’s coordinates to determine the position of each channel. The NIRS_SPM tool in MATLAB was then employed to convert these coordinates into MNI standard coordinates and to determine the coverage of the 37 channels across Brodmann areas (Table 1).

**Table 1:** MNI spatial location information for each fNIRS channel.

Channel	MNI coordinates			Brodmann area	Brain region	Coverage rate
	X	Y	Z			
1	61	1	45	6	Right pre–SMA	0.90152
2	47	22	50	8	Right Frontal eye fields	0.93333
3	26	36	55	8	Right Frontal eye fields	0.96761
4	0	38	55	8	Frontal eye fields	0.9697
5	–28	31	54	8	Left Frontal eye fields	0.98326
6	−49	11	50	8	Left Frontal eye fields	0.46154
7	−61	−16	46	6	Left pre–SMA	0.46099
8	68	−6	32	6	Right Frontal eye fields	0.77922
9	55	21	36	9	Right DLPFC	0.78777
10	32	53	36	9	Right DLPFC	0.79817
11	11	60	39	9	Right DLPFC	0.79921
12	−13	60	37	9	Left DLPFC	0.74902
13	−35	5	33	9	Left DLPFC	0.55752
14	−57	20	28	9	Left DLPFC	0.56641
15	−61	9	31	9	Left pre–SMA	0.57394
16	58	33	19	9	Right DLPFC	0.73038
17	49	38	31	9	Right DLPFC	0.6898
18	20	67	25	10	Right Frontopolar area	0.99635
19	−2	66	24	10	Frontopolar area	1
20	−24	66	23	10	Left Frontopolar area	1
21	−42	53	23	10	Left Frontopolar area	0.77586
22	−54	33	21	9	Left DLPFC	0.78049
23	60	29	6	45	Right Broca’s area	0.58689
24	50	51	8	9	Right DLPFC	0.45221
25	30	68	8	10	Right Frontopolar area	1
26	12	73	12	10	Right Frontopolar area	1
27	−14	73	10	10	Left Frontopolar area	1
28	−34	64	9	10	Left Frontopolar area	1
29	−48	48	10	9	Left DLPFC	0.61172
30	−59	23	11	45	Left Broca’s area	0.70794
31	54	45	−6	47	Right Inferior prefrontal gyrus	0.84932
32	38	65	−4	10	Right Frontopolar area	0.86142
33	20	72	−2	10	Right Frontopolar area	0.90064
34	−5	72	−1	10	Frontopolar area	0.87697
35	−23	70	−1	10	Left Frontopolar area	0.97342
36	−41	60	−3	10	Left Frontopolar area	0.89883
37	−54	40	−2	47	Left Inferior prefrontal gyrus	0.75000

Only brain regions with a coverage rate above 40% are listed.

### 2.4 Other measurements

An 11-point scale was employed to assess the level of attention focus and the anticipated analgesic effect (scores above 6).

### 2.5 Experimental design and procedure

This study adopted a 2 (experimental condition: expectancy group/control group) × 2 (time: pre-test/post-test) mixed experimental design, with experimental condition as a between-subjects variable and time (pre-test/post-test of pain intensity for test stimulus and pre-test/post-test of attention focus on test stimulus) as a within-subjects variable. The dependent variables were the pain score of test stimulus and the attention focus score on test stimulus.

The experimental procedure is as follows: after entering the laboratory, participants sign an informed consent form and then proceed with the formal experiment. An electrode cap is first placed on each participant to ensure proper signal quality across all channels. Capsaicin is applied to a 6 × 6 cm area on the inner side of the right ankle, which is then covered with plastic wrap for 20 min to facilitate heat generation and maintain a stable, continuous pain sensation (Li et al., 2020). After the 20-min period, participants rate the pain intensity on the inner side of the right ankle. To confirm effective induction of test stimulus, participants must report a moderate pain intensity to proceed; otherwise, they are excluded from the study. Eligible participants immerse their left hand in 12°C cold water for 1 min, after which they rate the pain intensity and attention focus level on the inner side of the right ankle within 3 min. The expectancy group receives verbal instructions (‘The water temperature will be automatically adjusted to achieve a good analgesic effect’), while the control group is informed that the water temperature remains unchanged. Both groups then undergo a second one-minute cold water immersion, after which participants rate the pain intensity, attention focus level, and anticipated analgesic effect on the inner side of the right ankle within 4 min. Finally, a 3D locator captures the channel position information. At the conclusion of the experiment, participants are compensated with 25 RMB, the expectancy group is debriefed on the true nature of the experiment, and psychological counseling is provided to all participants.

### 2.6 Statistical analyses

Data preprocessing, individual-level analysis, and 2D and 3D result visualization for fNIRS were performed using the NIRS_KIT data processing toolkit in MATLAB R2013b (Hou et al., 2021). Based on the simplest and most direct Bonferroni correction method within the FWE correction criteria (Oshina and Spigulis, 2021), the raw optical density data was converted into blood oxygen concentration changes. The accuracy of fNIRS signal data is often affected by interference noise and slow head movements of participants. To address this, a first-order polynomial regression model for detrending was applied in task-based analysis preprocessing to estimate linear and nonlinear trends, which were then subtracted from the original hemoglobin concentration signals. Temporal Derivative Distribution Repair was utilized to correct motion artifacts (Fishburn et al., 2019). For this study, an infinite impulse response (IIR) bandpass filter range of 0.01–0.018 Hz was applied to eliminate unrelated high- and low-frequency components. Individual-level analysis was performed using a general linear model (GLM), with the hemodynamic response function convolved to calculate *β* values as activation indices of oxygenated hemoglobin (HbO2) in the prefrontal cortex under different task conditions (Hall et al., 2021; Yeung et al., 2020).

Finally, 2 (experimental condition: expectancy group/neutral group) × 2 (time: pre-test/post-test) ANOVA was conducted on the β values for each channel before and after testing, using the statistical software SPSS 25.0.

## 3 Results

### 3.1 Behavioral results

**Figure 3:**
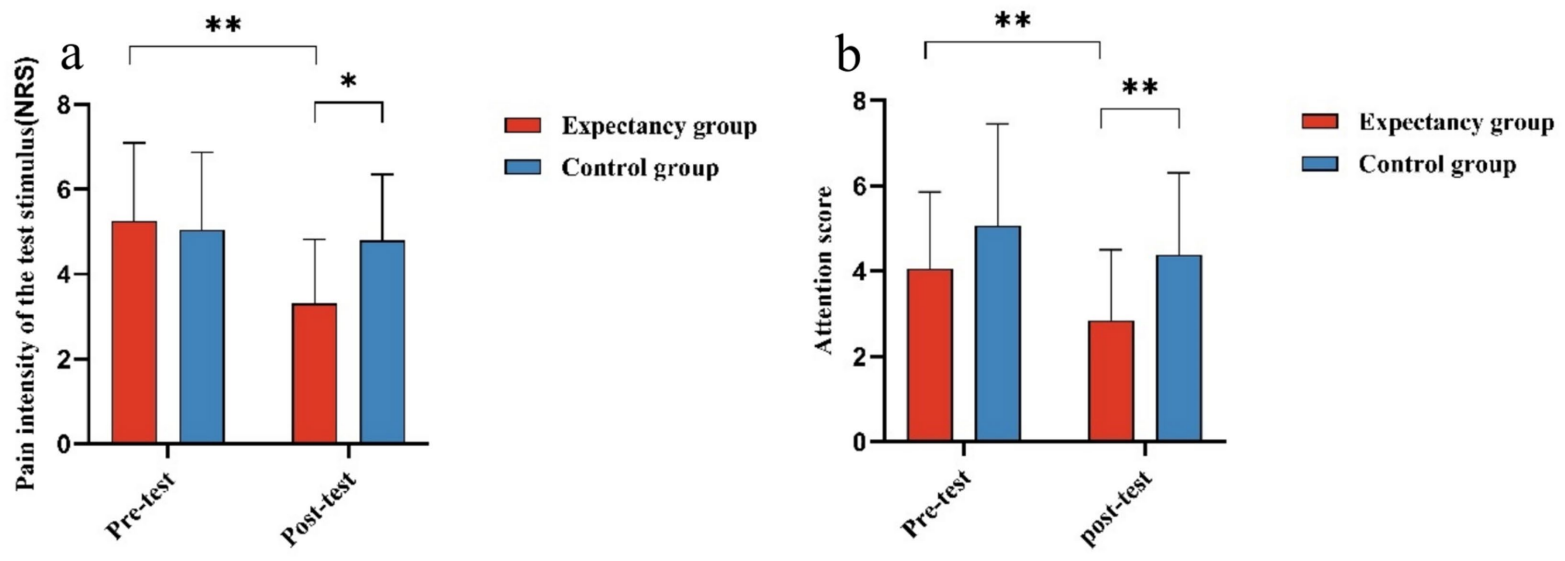
Analysis of variance for experimental conditions and time (pre- and post-assessment scores of experimental stimulus pain intensity and attention on the calf). **(a)** The difference in the pre- and post-assessment scores of experimental stimulus pain intensity perceived by the two groups of participants under different experimental conditions. The pain intensity of the experimental stimulus was measured using an 11-point scale ranging from 0 to 10, where 0 indicates no pain and 10 represents the most unbearable or imaginable pain. **(b)** The difference in the pre- and post-assessment scores of attention on the right ankle, as measured under different experimental conditions. The level of attention concentration on the right ankle was rated using the 11-point scale. *indicates *p* < 0.05, **indicates *p* < 0.01, and the error bars in the figure represent the standard error.

ANOVA for experimental condition groups and time (pre-test/post-test) revealed a significant interaction between experimental condition and pre/post-test scores of the test stimulus pain intensity, *F*(1, 37) = 4.725, *p* = 0.036, η^2^p = 0.113. Simple effects analysis indicated no significant difference between the control group’s pre-test score (4.926 ± 0.359) and post-test score (4.729 ± 0.421) for the test stimulus pain intensity. In contrast, the expectancy group’s post-test score (3.458 ± 0.410) was significantly lower than its pre-test score (4.283 ± 0.350), *F*(1, 37) = 16.758, *p* = 0.000, η^2^p = 0.312. The pre-test scores between the expectancy group (4.283 ± 0.350) and control group (4.926 ± 0.359) showed no significant difference; however, the control group’s post-test score (4.729 ± 0.421) was significantly higher than the expectancy group’s post-test score (3.458 ± 0.410), *F*(1, 37) = 4.685, *p* = 0.037, η^2^p = 0.112, as shown in Figure 3. ANOVA on experimental conditions and pre/post-test attention scores to test stimulus revealed a significant interaction between pre/post-test attention scores and experimental conditions, *F*(1, 37) = 9.383, *p* = 0.004, η^2^p = 0.202. Simple effects analysis showed no significant difference between the pre-test attention scores of the control group (5.061 ± 0.430) and the expectancy group (4.660 ± 0.419). However, the post-test attention score of the control group (4.595 ± 0.366) was significantly higher than that of the expectancy group (3.080 ± 0.357), *F*(1, 37) = 8.776, *p* = 0.005, η^2^p = 0.192. There was no significant difference between the control group’s pre-test (5.061 ± 0.430) and post-test attention scores (4.595 ± 0.366), whereas the expectancy group’s post-test attention score (3.080 ± 0.357) was significantly lower than its pre-test score (4.660 ± 0.419), *F*(1, 37) = 38.730, *p* = 0.000, η^2^p = 0.511, as shown in Figure 3.

Pearson correlation analysis revealed a significant positive correlation was observed between participants’ post-test pain intensity scores for the experimental stimulus and their attention post-test scores (*p* < 0.01, *r* = 0.884).

### 3.2 fNIRS results

**Figure 4:**
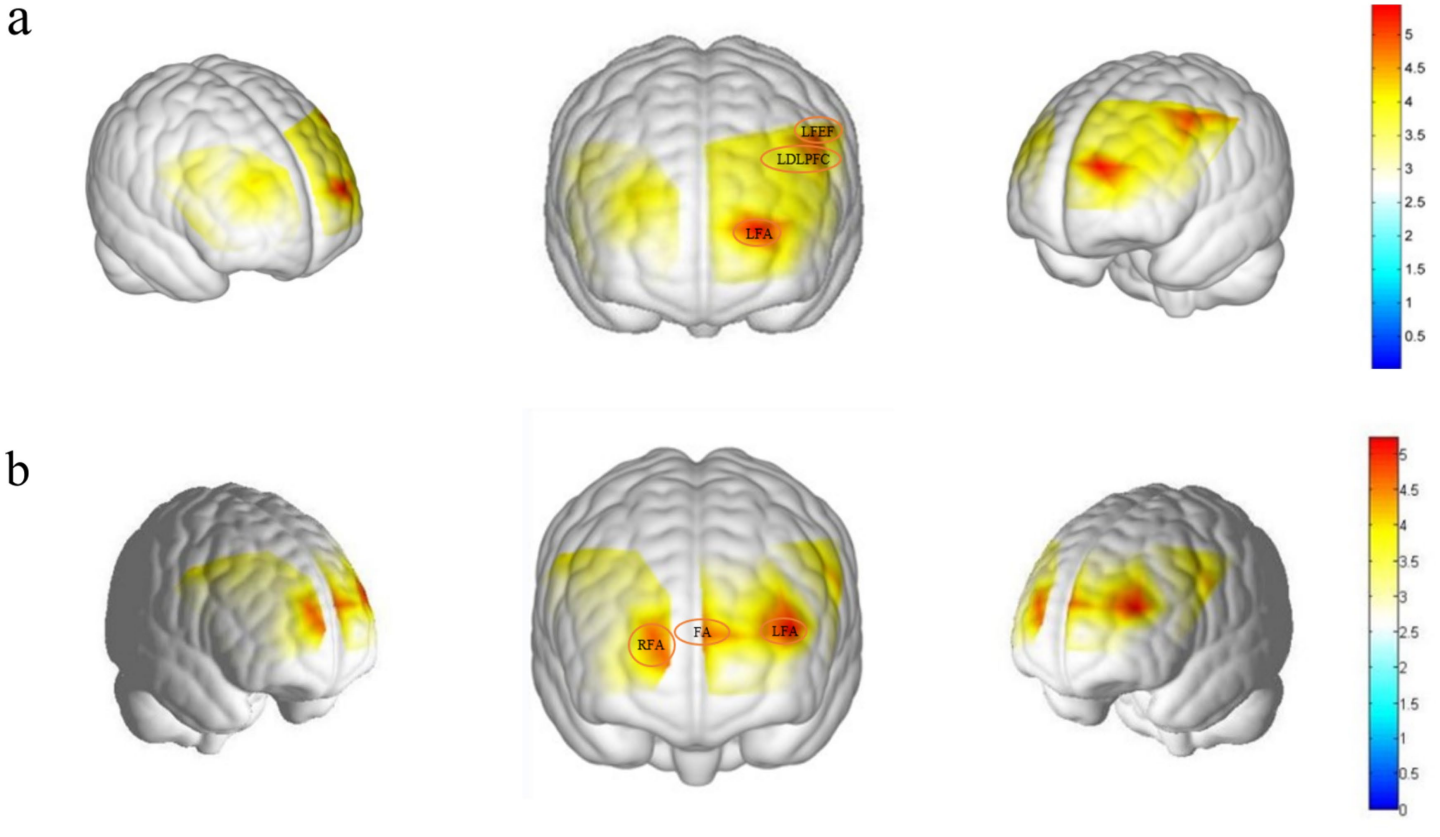
Brain region activation difference map. **(a)** The main effect map representing brain region activation (from left to right: right, anterior, left views), with involved brain regions being the Left Frontal eye fields (channel 6), the left DLPFC (channel 13), and the left frontopolar area (channel 20). **(b)** The interaction effect map representing brain region activation (from left to right: right, anterior, left views), with involved brain regions being the right frontopolar area (channel 18), the frontopolar area (channel 19), and the left frontopolar area (channel 21). The red areas within the borders indicate significant activation of these brain regions.

ANOVA on the *β* values for experimental conditions and pre/post-test times across 37 channels revealed significant main effects in the left frontal eye field (Channel 6), left dorsolateral prefrontal cortex (Channel 13), and left frontal pole (Channel 20), *F*(1, 37) = 4.389, *p* = 0.043, η^2^p = 0.106; *F*(1, 37) = 4.621, *p* = 0.038, η^2^p = 0.111; *F*(1, 37) = 5.454, *p* < 0.025, η^2^p = 0.128 (Figure 4). As presented in Table 2, *post-hoc* comparisons indicated that the activation levels in Channels 6, 13, and 20 were significantly lower in the post-test than in the pre-test.

**Table 2:** Main effect results for channels 6, 13, and 20.

Channel	Brain region	Channel pre-test β value (*M ± SD*)	Channel post-test β value (*M ± SD*)	*F*	p*	η^2^_p_
6	Left Frontal eye fields	−0.001 ± 0.001	−0.005 ± 0.001	4.389	0.043*	0.106
13	Left DLPFC	−0.003 ± 0.001	−0.006 ± 0.001	4.621	0.038*	0.111
20	Left Frontopolar area	−0.003 ± 0.001	−0.007 ± 0.001	5.454	0.025*	0.128

*indicates *p* < 0.05, **indicates *p* < 0.01, error bars indicate standard error.

The interaction between experimental conditions and the pre/post-test β values for Channels 18, 19, and 21 was significant, *F*(1, 37) = 4.136, *p* = 0.05, η^2^p = 0.106; *F*(1, 37) = 4.258, *p* = 0.046, η^2^p = 0.106; *F*(1, 37) = 5.222, *p* = 0.028, η^2^p = 0.124 (Figure 4).

Simple effects analysis indicated that in the control group, cortical activity intensity in the right frontopolar area (Channel 18) during the post-test (−0.002 ± 0.002) was significantly higher than that observed in the expectancy group (−0.007 ± 0.002). For the expectancy group, cortical activity intensity in the right frontopolar area (Channel 18) during the post-test (−0.007 ± 0.002) was significantly lower compared to the pre-test (−0.002 ± 0.002). Similarly, *post-test* activation levels in frontopolar area (Channel 19) for the expectancy group (−0.008 ± 0.002) were significantly lower than the pre-test levels (−0.001 ± 0.002). Additionally, in the expectancy group, cortical activation in the left Frontopolar area (Channel 21) during the post-test (−0.006 ± 0.002) was significantly lower than during the pre-test (0.001 ± 0.003).

Pearson correlation analysis revealed a significant positive correlation between Channel 7(Left pre–SMA) and participants’ post-test attention scores (*p* < 0.05, *r* = 0.347).

## 4 Discussion

### 4.1 The relationship between expectancy and CPM

By manipulating the experimental treatments for the expectancy and control groups, this study compared changes in pre- and post-test experimental stimulus pain intensity under different conditions, where lower pain intensity indicates a stronger CPM effect. Results showed that when positive expectancy was effectively induced, the expectancy group experienced more pronounced pain relief, resulting in a significantly greater CPM effect. This finding confirms that expectancy, as an intrinsic psychological mechanism, indeed plays a pivotal role in modulating CPM effects. The DLPFC, recognized as a key brain region in CPM modulation, is often associated with activation during expectancy-driven pain alleviation (Wager et al., 2011; Watson et al., 2009). However, this study revealed that under conditions of certain positive expectancy, the DLPFC exhibited notable deactivation. In fact, DLPFC activation depends on the certainty of the expectancy; uncertain expectancy is more likely to induce significant DLPFC activation than certain expectancy (Carlsson et al., 2006). Increased DLPFC signaling is, conversely, linked to reduced CPM capacity (Youssef et al., 2016). Moreover, a study on neuropathic pain treatment found that the analgesic effects of neuropharmacological agents were more closely related to deactivation, rather than activation, of the occipital and frontal lobes (Iannetti et al., 2005; Kong et al., 2007). Therefore, DLPFC deactivation supports the occurrence of CPM-induced analgesic effects.

### 4.2 The relationship between attention and CPM

While prior research has suggested that attention and CPM regulate pain through independent physiological mechanisms (Moont et al., 2012) and do not rely on the role of attention (Ladouceur et al., 2012), this study observed significant differences in post-test attention scores for test stimulus between the control and experimental groups, as well as significant pre- and post-test differences in attention scores within the expectancy group. These results indicate that attention may indeed play a meaningful role in CPM.

The perception of pain is regulated by top-down attentional mechanisms (Desimone and Duncan, 1995; Kastner and Ungerleider, 2000; Mouraux and Iannetti, 2009; Petersen and Posner, 2012). The prefrontal cortex, a critical part of the cerebral cortex, comprises regions such as the frontal eye fields, Frontopolar area, and dorsolateral prefrontal cortex (Fuster, 2001). It is implicated in diverse complex cognitive activities, including decision-making, working memory, and attentional regulation (Fuster, 2001; Miller and Cohen, 2001), with the left prefrontal region particularly essential for higher-order cognitive tasks. Studies employing the N-back paradigm to explore prefrontal structure and function have underscored the significance of the left prefrontal cortex in higher-level cognitive control (Volle et al., 2008). Additionally, functional magnetic resonance imaging (fMRI) studies on executive functions have confirmed that the left prefrontal cortex plays a role in attentional shifting (Sylvester et al., 2003). The frontal eye fields (FEF), part of the prefrontal cortex, are directly involved in the control of stimulus-driven attention (Corbetta and Shulman, 2002). While traditionally associated with visual attention and ocular movement control (Corbetta and Shulman, 2002), the FEF’s involvement in this study’s analysis of the relationship between CPM effects and attention demonstrates its role in modulating pain information, Undoubtedly, the changes in attention are intricately linked to the regulation by the SMA brain region (Borsook et al., 2013), The significant positive correlation between Channel 7 (Left pre–SMA) and the post-test attention scores further reinforces this connection, supporting the possibility that attention contributes to the regulation of pain within CPM-induced analgesia. The significant positive correlation between post-test attention and post-test TS pain intensity offers compelling evidence that reinforces the potential role of attention in modulating the CPM analgesic effect.

### 4.3 The relationship between expectancy and attention

Contemporary models of attention comprise both ‘top-down’ and ‘bottom-up’ selection mechanisms (Legrain et al., 2009). ‘Top-down’ selection represents a goal-directed, conscious cognitive process in which attention is unconsciously captured by stimuli (Failing and Theeuwes, 2018). This process enhances neural activity in response to relevant stimuli while suppressing activity related to irrelevant stimuli, thereby modulating the sensitivity of stimulus-specific neural responses (Desimone and Duncan, 1995).

Pain is recognized as a ‘bottom-up’ control mechanism that automatically draws attention through warning signals, disrupting ongoing activities (Eccleston and Crombez, 1999; Van Damme et al., 2004) and prioritizing protective behaviors to avert bodily harm (Legrain et al., 2011). This indicates that ‘bottom-up’ attention to pain can be modulated through ‘top-down’ mechanisms. In pain management, ‘top-down’ regulation of attention using distraction tasks has demonstrated efficacy in reducing pain perception (Johnson, 2005; Kohl et al., 2013). For example, engaging participants in highly demanding visual tasks reduces the amplitude of the P2 component of nociceptive-evoked potentials (Legrain et al., 2005). Expectancy, as a proactive coping strategy initiated by the brain before the onset of a stimulus, influences the allocation of attentional resources (Atlas and Wager, 2012; Desimone and Duncan, 1995; Summerfield and Egner, 2009). The frontal pole is not only involved in complex task-related decision-making and expectancy (Koechlin and Hyafil, 2007) but also plays a role in attentional regulation (Irani et al., 2007). This study revealed significant behavioral differences in pre- and post-test attention to experimental stimuli between the expectancy and control groups, as well as variations in blood oxygen changes in the left and right frontal poles detected via fNIRS. The marked changes in blood oxygen concentration in the frontal pole may suggest that expectancy processing of pain information within the CPM effect facilitated shifts in attention away from test stimulus pain. This implies that the psychological mechanism of the CPM effect is driven by expectancy through a ‘top-down’ process, influencing analgesic outcomes by modulating the allocation of attention.

### 4.4 Changes in the PFC within CPM

The PFC serves as a central component of the brain’s cognitive control network (Badre, 2008), with distinct functional differences between the left and right PFC. The left PFC predominantly processes the meaning and features of individual events, facilitates rapid event activation, and strongly inhibits adjacent events. In contrast, the right PFC emphasizes cross-temporal and spatial integration of event meanings and characteristics, engages in slower event activation, and exhibits weaker facilitation of neighboring events (Wood and Grafman, 2003). Research using alpha brainwave activity to examine lateralization effects in the PFC has shown that the left PFC supports the behavioral activation system (BAS), while the right PFC underpins the behavioral inhibition system (BIS; Coan and Allen, 2003). BAS is linked to reward pursuit and positive behavior, functioning as a motivational system that drives individuals toward desired outcomes or potentially rewarding stimuli (Kennis et al., 2013). In contrast, BIS is described as a system that interrupts ongoing behavior, heightens arousal, and increases attentional focus in response to potential punishment, novel stimuli, or uncertain outcomes (Gray, 1994; Corr, 2004). Furthermore, the lateralization of the PFC is evident in emotion processing, with the left PFC primarily regulating positive emotions, while the right PFC specializes in processing negative emotions (Demaree et al., 2005). Activation of the BAS system promotes approach behaviors and elicits positive emotional experiences, whereas deficits in the BAS system increase the risk of depression (McFarland et al., 2006). Conversely, activation of the BIS system is associated with negative emotions, such as anxiety (Li et al., 2008).

The fNIRS results from this study indicated that changes in brain activity were predominantly localized to the left PFC regions, specifically the left frontal eye field, left dorsolateral prefrontal cortex, and Left Frontopolar area. This highlights the prominent role of the left PFC in pain regulation. During experimentally induced pain, increased activity in the left PFC correlates with pain perception, and when pain is alleviated due to motivational drives for pain relief rewards, activity in the left PFC decreases (Silva Passadouro et al., 2022). This suggests that inducing a clear analgesic expectancy effectively activates reward-seeking motivation (Fields, 2007), leading to reduced levels of oxygenated hemoglobin in the left PFC and the suppression of right PFC activity in pain modulation. Supporting evidence includes studies demonstrating that right PFC oxygenated hemoglobin activity diminishes during expectancy-induced pain relief (Silva Passadouro et al., 2022).

### 4.5 Psychological mechanism of expectancy in modulating CPM

Pain is not merely a direct interpretation of noxious stimuli; rather, it represents a complex interplay of sensory, emotional, motivational, and cognitive experiences. This protective function fosters motivation and learning, encoded by the brain’s reward-motivation cortical circuits that register both the aversive aspects of pain and its relief (Navratilova and Porreca, 2014). These circuits encompass regions such as the prefrontal cortex, ventral tegmental area, amygdala, and hypothalamic (Haber and Knutson, 2010; Navratilova and Porreca, 2014; Schultz, 2000). Positive expectancy activates reward-seeking motivations, while negative expectancy triggers avoidance responses (Fields, 2007). Our findings also confirmed that when analgesic expectancy was clearly established, participants’ BAS system exhibited significant activation. This activation of positive expectancy aligns reward-seeking motivations with the drive to avoid pain, supported by neuroimaging evidence showing activation in the left PFC.

Moreover, the allocation of attention to information is shaped by motivation (Di Nocera et al., 2014) and modulated by emotional states, with their interplay influencing an individual’s subjective pain experience (Villemure and Schweinhardt, 2010). Studies in China have also established a significant correlation between heightened attention to pain and increased negative emotions (Liu, 2024). Positive and negative emotional states drive individuals to engage in escape or approach behaviors, while allowing them to learn and anticipate whether future circumstances will be threatening or beneficial (Wiech and Tracey, 2013).

**Figure 5:**
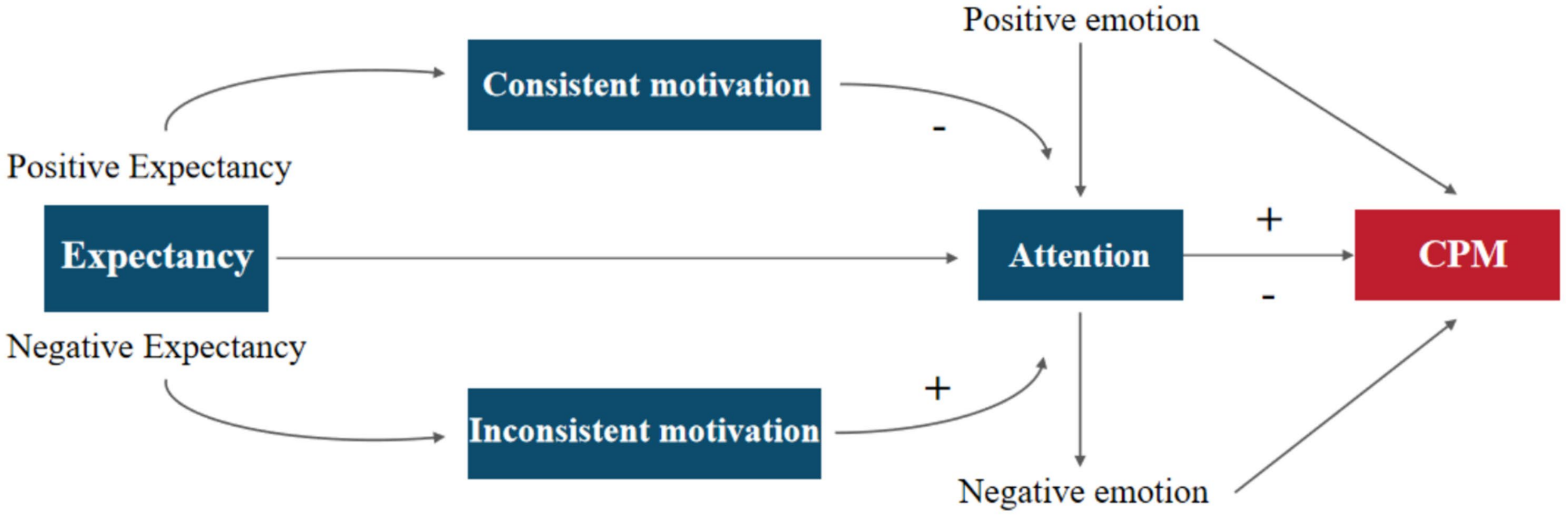
EACM model, where “–” indicates a decrease and “+” indicates an increase.

According to the Cyclical Model of Pain, Executive Function, Emotion Regulation, and Self-Management (the COPES model), there exists a cyclical relationship among immature executive function and emotional regulation, reduced self-management capability, chronic pain experiences, and increased pain-related disability (Caes et al., 2021). Chinese scholars have suggested that individuals can learn to reduce negative experiences, thereby updating the perceived meaning of pain and fostering positive expectancy for future pain episodes (Liu et al., 2022). This theory provides new perspectives on the psychological mechanisms by which CPM alleviates pain. Researchers have proposed the Expectancy-Attention-CPM Modulation Model (EACM Model), depicted in Figure 5.

According to the EACM model: During the process of the CPM effect, individuals can learn to manage their pain expectancy through self-regulation. When positive expectancies are formed, they activate the individual’s positive motivational system, generating analgesic motivations aligned with pain avoidance. This influences attentional resource allocation, shifting focus away from pain, thereby reducing pain perception and enhancing the CPM analgesic effect. In contrast, negative expectancies elicit conflicting motivations that are inconsistent with pain avoidance, leading to increased attention to pain and heightened pain perception, ultimately weakening the CPM effect. Moreover, observed changes in PFC activity are closely linked to emotional regulation (Etkin et al., 2015). Hence, within this framework, emotions emerge as another psychological factor affecting CPM efficacy. Emotions can interact with attention to directly shape pain experiences or exert direct influence on the CPM effect. For example, negative emotional states such as pain catastrophizing correlate with reduced pain modulation capacity, where higher levels of pain catastrophizing are associated with weaker pain regulation and lower CPM efficiency (King et al., 2013).

### 4.6 Limitations

This study utilized two distinct pain induction methods. The first method involved applying capsaicin to the body to induce heat pain as an experimental paradigm, offering a highly reproducible form of stimulation. The second method employed was the cold pressor test (CPT), a widely used and effective pain induction paradigm in laboratory settings, in which participants immerse their hands in cold water up to the wrist. Researchers used 12°C water to elicit pain in participants, effectively serving as a conditioned stimulus. However, due to the relatively slow temperature regulation of cold-water apparatuses, precise control over the intensity of the conditioned stimulus remains challenging. Future studies should explore alternative induction methods, such as laser or electrically induced pain stimuli. Moreover, pain and conditioned pain modulation, as experiences and regulatory processes with emotional dimensions, involve not only cortical structures like the DLPFC but also deeper nuclei such as the periaqueductal gray (PAG) and the anterior cingulate cortex (ACC; Yarnitsky, 2010). Regrettably, due to the inherent limitations of fNIRS (Scholkmann et al., 2014), this study was confined to observing cortical activity, thereby precluding the investigation of brain activity in deeper structures implicated in conditioned pain modulation. This limitation may have constrained the depth of the findings. Future research could integrate fMRI, TMS, and other technologies to further investigate additional brain regions associated with pain processing, offering a more comprehensive view of the neural mechanisms underlying CPM-induced analgesia.

Laboratory conditions differ substantially from clinical settings. The ecological validity of the conclusions derived from this study in the laboratory—regarding their potential application in clinical practice—remains to be further examined. Furthermore, as the participants in this study were all healthy adults, it is crucial to explore whether these findings can be generalized to other populations, such as chronic pain sufferers or the elderly. Future research should investigate the psychological and neural mechanisms involved in pain processing during conditioned pain modulation across diverse populations, thereby improving the ecological validity of this study.

The expectancy manipulation in this study was induced through verbal guidance, potentially subject to individual differences such as personality traits, which could lead to varying degrees of effectiveness in the expectancy group. Furthermore, the expectancy approach utilized may involve elements of deception. Although participants provided informed consent prior to the experiment and received full debriefing afterward, this process may still raise concerns about infringing upon their right to informed consent. Thus, minimizing or avoiding deception remains a pressing issue. Researchers have proposed the use of an ‘open-label’ approach, which may serve as a means to induce expectancy effects without deception. This involves enlisting participants who have previously undergone expectancy induction to facilitate expectancy effects in current participants (Charlesworth et al., 2017). Nevertheless, whether the ‘open-label’ approach can truly avoid deception warrants further investigation.

## 5 Conclusion

This study employed a CPM induction paradigm with capsaicin-induced pain as the test stimulus (TS) and a fixed-duration cold-water immersion as the conditioning stimulus (CS). The findings underscored that expectancy serves as a pivotal psychological mechanism in CPM, with “top-down” expectancy amplifying the analgesic effects of CPM. Moreover, the study identified a potential role for attention in modulating CPM effects, suggesting that expectancy may regulate CPM through attention control. To further elucidate these mechanisms, the researchers introduced the ECMA model, aiming to describe the psychological dynamics of CPM and offer innovative perspectives for its clinical application, ultimately benefiting patients experiencing pain.

## Data Availability

The raw data supporting the conclusions of this article will be made available by the authors, without undue reservation.
